# The pattern of late gadolinium enhancement by cardiac MRI in fulminant myocarditis and its prognostic implication: a two-year follow-up study

**DOI:** 10.3389/fcvm.2023.1144469

**Published:** 2023-06-27

**Authors:** Luying Jiang, Houjuan Zuo, Jingbo Liu, Jianyu Wang, Kaiyue Zhang, Chunran Zhang, Xiangyang Peng, Yujian Liu, Daowen Wang, Haojie Li, Hong Wang

**Affiliations:** ^1^ Division of Cardiology, Department of Internal Medicine, Tongji Hospital, Tongji Medical College, Huazhong University of Science and Technology, Wuhan, China; ^2^ Hubei Key Laboratory of Genetics and Molecular Mechanisms of Cardiologic Disorders, Wuhan, China; ^3^ The 3rd Department of Cardiology, The First Affiliated Hospital of The Medical College, Shihezi University, Shihezi, China; ^4^ Department of Radiology, Tongji Hospital, Tongji Medical College, Huazhong University of Science and Technology, Wuhan, China

**Keywords:** fulminant myocarditis, late gadolinium enhancement, global longitudinal strain, left ventricular function, follow-up study

## Abstract

**Background:**

Myocardial fibrosis, as quantified by late gadolinium enhancement (LGE) in cardiac magnetic resonance (CMR), provides valuable prognostic information for patients with myocarditis. However, due to the low incidence rate of fulminant myocarditis (FM) and accordingly small sample size, the knowledge about the role of LGE to patients with FM is limited.

**Methods and results:**

A total of 44 adults with viral-FM receiving the Chinese treating regimen were included in this retrospective study. They were divided into the low LGE group and the high LGE group according to the ratio of LGE to left ventricular mass (LGE mass%). CMR exams and LGE were performed after hemodynamic assistance at discharge in all patients with FM. Routine echocardiography parameters and global longitudinal strain (GLS) at discharge and at 2-year follow-up were obtained and then compared. Both left ventricular ejection fraction (LVEF) and GLS showed no significant difference in both groups at discharge, whereas significant differences were observed at 2-year follow-up between two groups. Moreover, there were significant improvements of LVEF and GLS in the low LGE group, but not in the high LGE group during the 2-year period. Furthermore, LGE mass% was negatively correlated with GLS and LVEF.

**Conclusions:**

There were two distinct forms of LGE presentation in patients with FM. Moreover, the cardiac function of patients with low LGE was significantly better than those with high LGE at 2-year follow-up. LGE mass% at discharge provided significant prognosis information about cardiac function of patients with FM.

## 1. Introduction

Myocarditis is a condition that causes ventricular systolic dysfunction due to inflammation of the myocardium. It appears to be a major cause of sudden cardiac death in patients under the age of 40 (1). Myocarditis is classified into fulminant myocarditis (FM) or non-fulminant acute myocarditis (NFAM) based on clinical presentations, histological features, echocardiographic index, and hemodynamic stability (2). FM, however, is the most severe type of myocarditis characterized by acute, severe heart failure. Patients with FM are often in cardiogenic shock and require immediate mechanical circulatory support (MCS) (3). Even managed with aggressive pharmacological therapy and mechanical support, the in-hospital mortality rate of FM is considerably high (up to 40%–60%) (4, 5). Our center recently raised the Chinese protocol in treating FM termed as life support-based comprehensive treatment regimen (LSBCTR), which has been proved to reduce mortality rate to <5% (6, 7).

Endomyocardial biopsy (EMB) represents the golden standard for the diagnosis of FM (8). However, it is limited by possible sampling errors and its inherent procedural risk of invasive examination (9–12). Cardiac magnetic resonance (CMR), with the advantages of detecting signals of myocardial damage such as myocardial edema, hyperemia, and fibrosis (13, 14), has been applied in the diagnosis and prognosis evaluation of myocarditis. Among them, late gadolinium enhancement (LGE) can identify areas of fibrotic myocardium that are closely related to ventricular arrhythmias (15). Recently, there has been an increasing interest in the prognostic value of LGE in myocarditis for possible adverse clinical outcomes. Grun et al. showed that LGE was the best independent predictor of all-cause mortality and cardiac mortality in patients with a wide range of clinical symptoms and biopsy-proven viral myocarditis (16). Recently, LGE has been shown to have additive prognostic value in stratifying risk of patients with suspected myocarditis (14). Additionally, Barone-Rochette et al. reported a trend of a worse outcome in patients with suspected myocarditis with higher LGE extent scores (17). Nevertheless, there are limited data (18–20) with relatively small sample sizes focusing on the value of LGE in FM. As a result, the role of LGE in patients with FM remains unknown. Does the pattern of LGE in patients with FM have any relationship with the cardiac function or prognosis of these patients? Therefore, the aim of this study was to assess the pattern of LGE in patients with FM and the correlation between LGE and cardiac function at 2-year follow-up.

## 2. Method

### 2.1. Study population

A total of 97 patients with clinically suspected acute myocarditis were admitted to the Tongji Hospital, Tongji Medical College, Huazhong University of Science and Technology, the largest myocarditis center in China between October 2017 and February 2021. The diagnosis of NFAM was made on the basis of clinical evidence and two or more CMR Lake Louise criteria (myocardial edema, hyperemia, and LGE). Endomyocardial biopsy was performed when CMR results were inconclusive (<1 CMR criterion). For clinically unstable FM patients, however, CMR exams may be delayed and performed after hemodynamic assistance.

Relevant clinical data were retrieved from the patients’ electronic medical records. All patients with NFAM presented acute chest pain, elevated troponin, and a history of viral infection with less than 2 weeks. Exclusion criteria were: (1) prior history of coronary artery disease (CAD), myocardial infarction, or other acute or chronic cardiac diseases; (2) contraindications for CMR or fatal arrhythmias.

**Figure 1:**
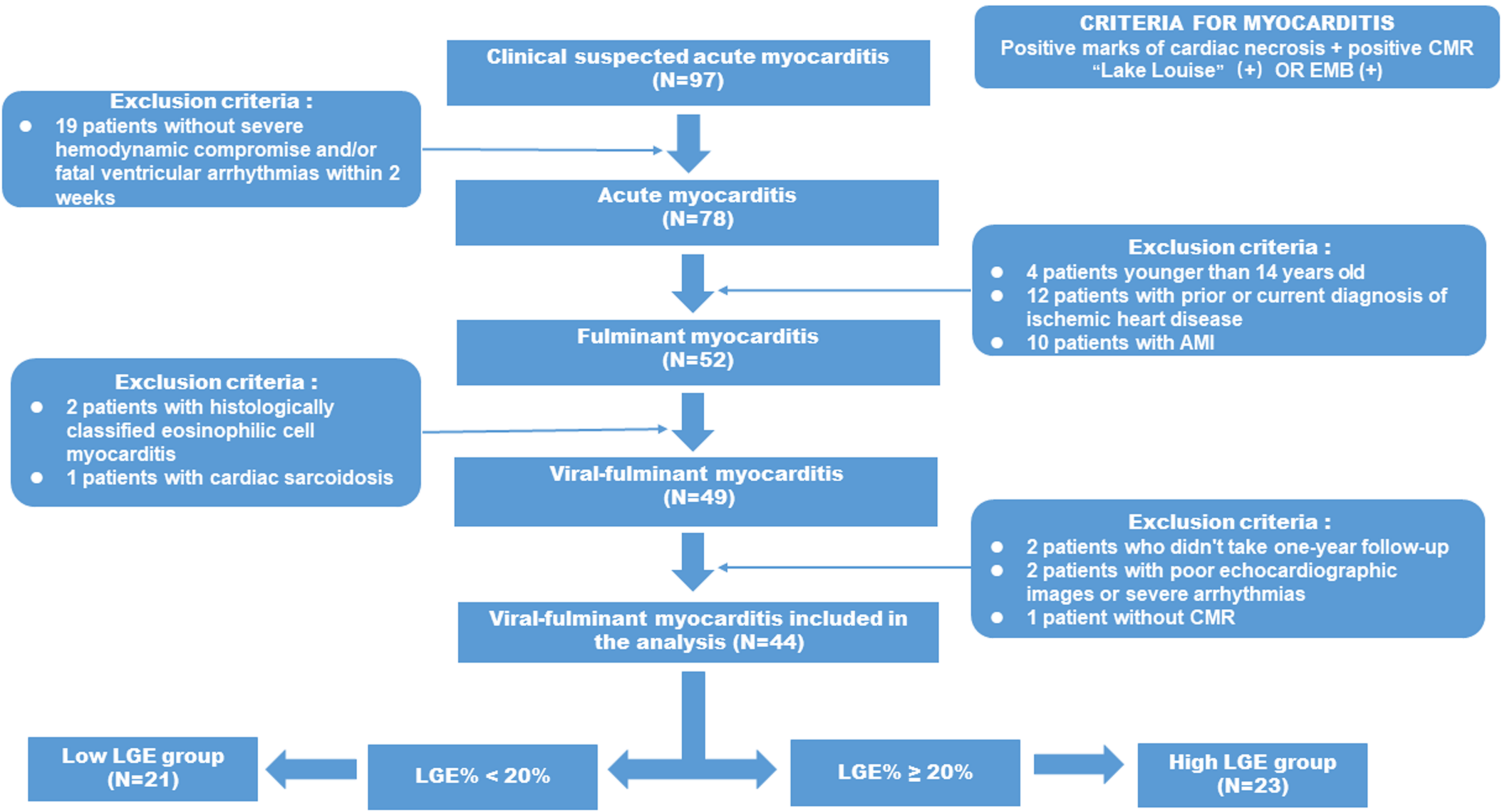
Flow diagram describing the enrolling patients and selection protocol of high LGE group and low LGE group adult patients with FM from the 97 patients with suspected AM were screened initially in the present study and the final study population consisted of 21 FM patients in the low LGE group and 23 FM patients in the high LGE group.

Invasive coronary angiography was performed on all patients, except those younger than 35 years of old with low risk of coronary artery disease. Fulminant myocarditis was defined as acute myocarditis patients requiring inotropic drug and/or MCSs (21). The primary analysis of this study focused on viral myocarditis, defined as patients with significant viral infection-like prodromal symptoms, while patients with EMB-proven eosinophilic myocarditis were excluded. The detailed study workflow is shown in Figure 1.

The final population included 44 viral-FM patients who completed the 2-year follow-up. They were divided into high LGE group (*n* = 23) and low LGE group (*n* = 23) according to whether the left ventricular mass (LGE mass%) >20. Informed consent was obtained from all patients at the time of the CMR examination.

### 2.2. Patient management

The LSBCTR was applied to all FM patients enrolled in the study. The vital of this regimen is the early administration of MCS to provide circulatory support. At our center, we prefer to use intra-aortic balloon pump (IABP) as first-line MCS approach (3). In cases where hemodynamic disturbances cannot be corrected after using IABP, venoarterial extracorporeal membrane oxygenation (VA-ECMO) was added in combination with IABP. Once stabilized, all FM patients with systolic dysfunction were treated by evidence-based heart failure therapy.

After the CMR examination, all included patients with FM were followed up by the investigators. All enrolled patients receive routine echocardiography examination during the 2-year follow-up, and patients who had been lost within 2 years and patients with poor echocardiography image quality were excluded.

### 2.3. Conventional echocardiographic examination and strain analysis

Conventional echocardiography was performed using a single commercially echocardiographic system (Vivid E9; GE Vingmed, Horten, Norway) equipped with a 3.5 MHz transducer.

The echocardiographic records were obtained and analyzed by a professional cardiologist experienced in echocardiographic analysis at the time of admission, before discharge, and 2-year follow-up, respectively.

Left ventricular ejection fraction (LVEF) was calculated by the modified biplane Simpson's method from apical four-chamber and two-chamber views, and averaged for continuous three beats. Other conventional parameters including the left atrial (LA) diameter, left ventricular end-diastolic dimensions (LVEDD), internal diameter and LV diastolic hemodynamic indices were measured based on the American Society of Echocardiography criteria and the European Association of Cardiovascular Imaging (22).

**Figure 2:**
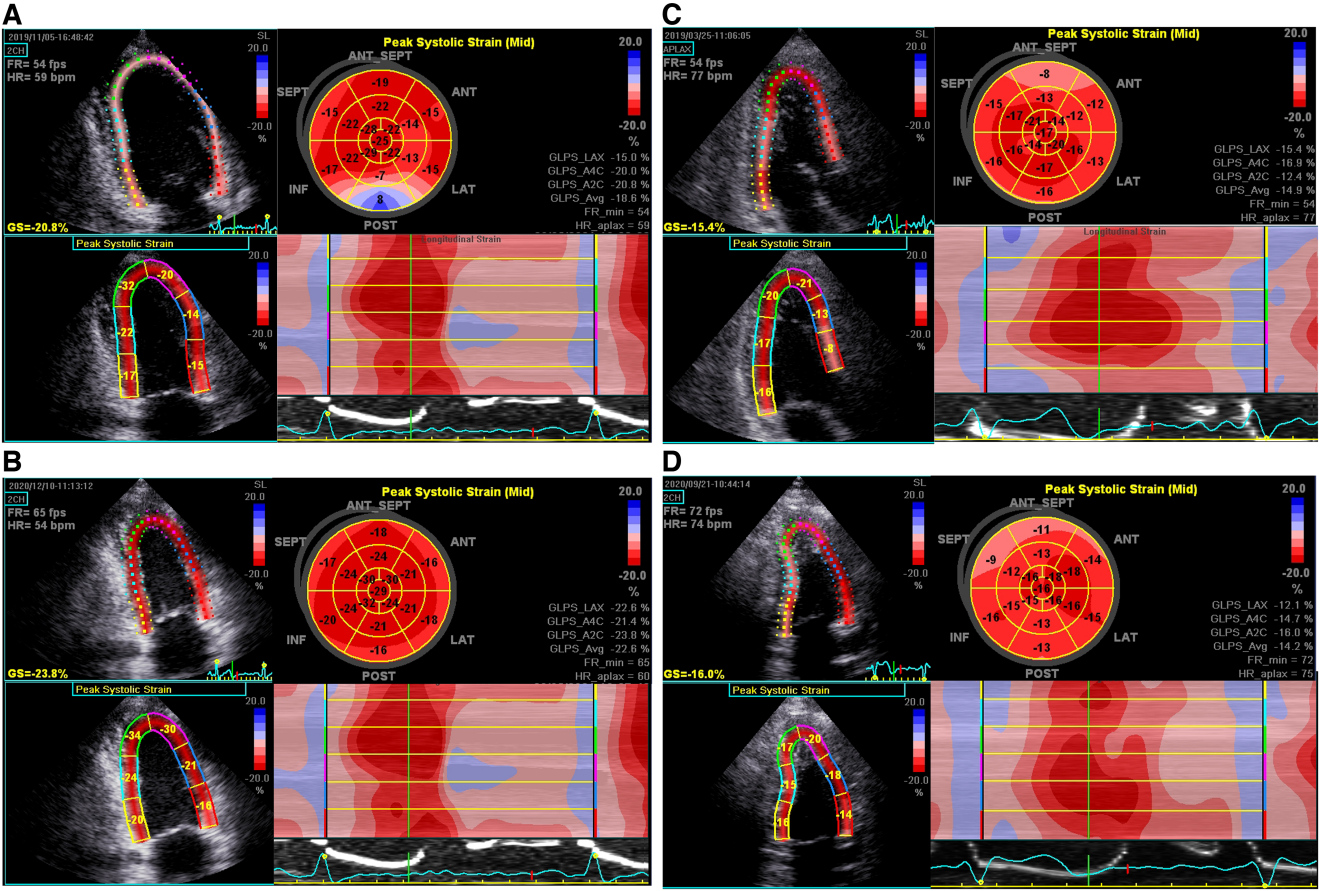
Representative bull's-eye displays of GLSs in a 30-year-old man (**A,B**) and a 39-year-old man (**C,D**) with FM, respectively, upon discharge and 2-year follow-up. (**A**) Significant reduction of PSLSs in basal-, mid-inferior, posterior, and antero-septal segments, with the average GLS being 18.6%; (**B**) complete normalization of GLS; (**C**) significant reduction of PSLSs in basal-, mid-, apical-inferior, posterior and antero-septal segments at discharge, with the average GLS being 14.9%; (**D**) the GLS remained at 14.2%.

Global longitudinal strain (GLS) was obtained by two-dimensional (2D) speckle tracking echocardiography (STE). The 2D gray scale images of three consecutive cardiac cycles for each of the apical two-chamber, three-chamber, and four-chamber views at end-systole were saved for strain analysis. Data processing was conducted offline by using Echo Pac (version: 113, 2017; GE Vingmed, Horten, Norway). GLS was quantified by automated functional imaging analysis, based on the AHA 17-segment LV model, expressed as the absolute value of the mean. Representative images of “bull’s-eye” of two FM patients with different LGE mass% were showed in Figure 2.

### 2.4. CMR acquisition and analysis

CMR exams were delayed and performed in all FM patients after hemodynamic assistance by a 3T MRI scanner (Magnetom Skyra, Siemens Healthcare, Erlangen, Germany) using an 18-channel body matrix coil. All cardiac MR images were assessed offline by two independent blinded readers with 2 or more than 2 years of experience who were blinded to the clinical and echocardiographic data of the patients, using commercial cardiac software cvi42 (v. 5.3, Circle Cardiovascular Imaging, Calgary, Canada).

LGE imaging was performed by using segmented inversion-recovery gradient-echo sequences at the horizontal long axis, the vertical long axis, and the short axis as previously described (23). The area of visually apparent LGE from the mitral valve to the LV apex were summed to generate total volume of LGE in grams and calculated its percentage to total LV myocardial mass. The manual LGE quantitation method has been shown to be the best to histopathologically reflect total fibrosis burden (24). LGE mass% was defined as the percentage of LGE mass to total LV mass.

### 2.5. Statistical analysis

Statistical analysis was performed by using software (SPSS 20.0, Chicago, IL, United States). Continuous variables were expressed as mean ± SD or as median and interquartile range (IQR), depending on the normality of distribution. The Shapiro–Wilk test was performed to test the normality of the variables. Continuous variables between groups were analyzed by Student’s *t*-test or Mann–Whitney *U* test, depending on the normality of the variables. Categorical variables were evaluated with square test or Fisher exact test. Relationships among multivariables were analyzed by Pearson correlation analysis.

*P*-value lower than 0.05 was considered statistically significant.

## 3. Result

### 3.1. Population Characteristics

Baseline characteristics of the entire population and groups are summarized in Table 1. A total of 44 patients were enrolled in this study (21 patients with low LGE and 23 with high LGE). All patients with FM received an intra-aortic balloon pump, while 10 patients further received VA-ECMO treatment, of which 5 were in the low LGE group. The data of FM patients at admission are shown in Supplementary Table S1.

**Table 1:** Characteristics of the low LGE and high LGE groups at discharge.

	Low LGE	High LGE	*P*-value
Male - *n* (%)	11 (52.4)	10 (43.5)	0.555
Female - *n* (%)	10 (47.6)	13 (56.5)	0.555
Age (year)	33.71 ± 12.48	31.83 ± 11.46	0.604
Height (cm)	166.29 ± 9.25	167.17 ± 6.48	0.717
Weight (kg)	68.19 ± 16.33	61.67 ± 10.71	0.121
Body surface area (m^2^)	0.73 ± 0.21	0.65 ± 0.14	0.122
Body mass index (kg/m^2^)	24.37 ± 3.86	21.98 ± 2.96	0.026
Systolic blood pressure (mmHg)	115.86 ± 8.69	108.48 ± 12.09	0.029
Diastolic blood pressure (mmHg)	72.95 ± 9.30	65.52 ± 8.54	0.01
Heart rate (bpm)	75.48 ± 10.97	72.81 ± 10.18	0.419
Biochemistry and urinary examination			
CRP (mg/L)	12.50 (4.43–25.45)	6.10 (2.18–19.38)	0.052
Peak hs-TNT (pg/ml)	16,670.50 (6,547.53–42,998.75)	50,000.00 (36,101.60–50,000)	0.001
hs-TNT (pg/ml)	93.15 (54.70–255.73)	166.70 (100.55–466.58)	0.076
NT-proBNP (pg/ml)	395.00 (174.25–806.00)	667.00 (435.50–1,220.50)	0.012
ALT (U/L)	47.00 (33.00–77.50)	59.00 (40.00–75.25)	0.789
AST (U/L)	43.5017 (50.00–67.00)	50.00 (21.50–86.75)	0.238
Creatinine (μmol/L)	74.00 (69.00–90.00)	63.50 (52.25–71.00)	0.006
Lactic acid (mmol/L)	1.65 (1.14–3.25)	1.68 (1.33–2.72)	0.907
Glucose (mmol/L)	7.76 ± 2.32	8.00 ± 2.92	0.766
Clinical presentation			
Chest distress, *n* (%)	11 (52.4)	18 (78.3)	0.07
Chest pain, *n* (%)	10 (47.6)	10 (43.5)	0.783
Dyspnea, *n* (%)	5 (23.8)	7 (30.4)	0.622
Fever, *n* (%)	10 (47.6)	13 (56.5)	0.555
Vomit, *n* (%)	4 (19)	6 (26.1)	0.844
Diarrhea, *n* (%)	2 (9.5)	1 (4.3)	0.935
Associated autoimmune disorders, *n* (%)	3 (14.3)	6 (26.1)	0.552
Arrhythmology, *n* (%)	15 (71.4)	21 (91.3)	0.188
ECG			
T-wave inversion, *n* (%)	3 (14.3)	8 (34.8)	0.117
ST-segment elevation, *n* (%)	4 (19)	13 (56.5)	0.011
Other abnormal ST-T segment, *n* (%)	13 (61.9)	11 (47.8)	0.349
Ventricular arrhythmia, *n* (%)	11 (52.4)	12 (52.2)	0.989
Bundle branch block, *n* (%)	7 (33.3)	7 (30.4)	0.837
III atrioventricular block, n (%)	2 (9.5)	4 (17.4)	0.749
Treatment			
IABP, *n* (%)	21 (100)	23 (100)	
Days of IABP use (day)	5.00 (5.00–6.50)	4.00 (1.50–8.25)	0.261
ECMO, *n* (%)	5 (23.8)	5 (21.7)	1
Days of ECMO use (day)	0.00 (0.00–1.50)	0.00 (0.00–0.75)	0.831
CVVH, *n* (%)	9 (42.9)	5 (21.7)	0.133
Days of CVVH use (day)	0.00 (0.00–1.00)	0.00 (0.00–0.00)	0.126
Glucocorticoid (mg)	800.00 (690.00–1,085.00)	850.00 (515.00–1,057.50)	0.799
Days of glucocorticoid use (day)	8.00 (6.00–9.00)	7.00 (6.00–10.00)	1
R-globulin (g)	60.00 (50.00–75.00)	33.75 (55.00–65.00)	0.137
Days of r-globulin use (day)	7.00 (6.00–7.00)	6.00 (3.75–7.00)	0.148
Post-discharge medication			
Glucocorticoid, *n* (%)	12 (57.1)	16 (69.6)	0.392
ACEI/ARB, *n* (%)	17 (81)	13 (56.5)	0.082
β-blocker, *n* (%)	17 (81)	17 (73.9)	0.844
MRA antagonists, *n* (%)	0 (0)	1 (4.3)	1
SGLT2 inhibitors, *n* (%)	1 (4.76)	2 (8.70)	0.601

CRP, C-reactive protein; ALT, alanine aminotransferase; AST, aspartate aminotransferase; IABP, intra-aortic balloon pump; ECMO, extracorporeal membrane oxygenation; CVVH, continuous venovenous hemofiltration; ACEI/ARB, angiotensin-converting enzyme inhibitor or angiotensin receptor blocker.

Age (*P* = 0.604) or sex (*P* = 0.555) did not differ significantly between the two groups. Peak level of high-sensitivity cardiac troponin T (hs-TNT) [50,000.00 (36,101.60–50,000) ng/ml vs. 16,670.50 (6,547.53–42,998.75) ng/ml, *P* = 0.001] and the level of N-terminal pro–B-type natriuretic peptide (NT-proBNP) at discharge [667.00 (435.50–1,220.50) pg/ml vs. 395.00 (174.25–806.00) pg/ml, *P* = 0.012] of the high LGE group was significantly higher than those of the low LGE group. There was no significant difference in ECG abnormalities between the two groups, with the exception that patients with ST-segment elevation who were more likely to be present in the high LGE group (13 of 23, 56.5%, vs. 4 of 21, 19%; *P* = 0.011).

### 3.2. Cardiac MRI Results

**Figure 3:**
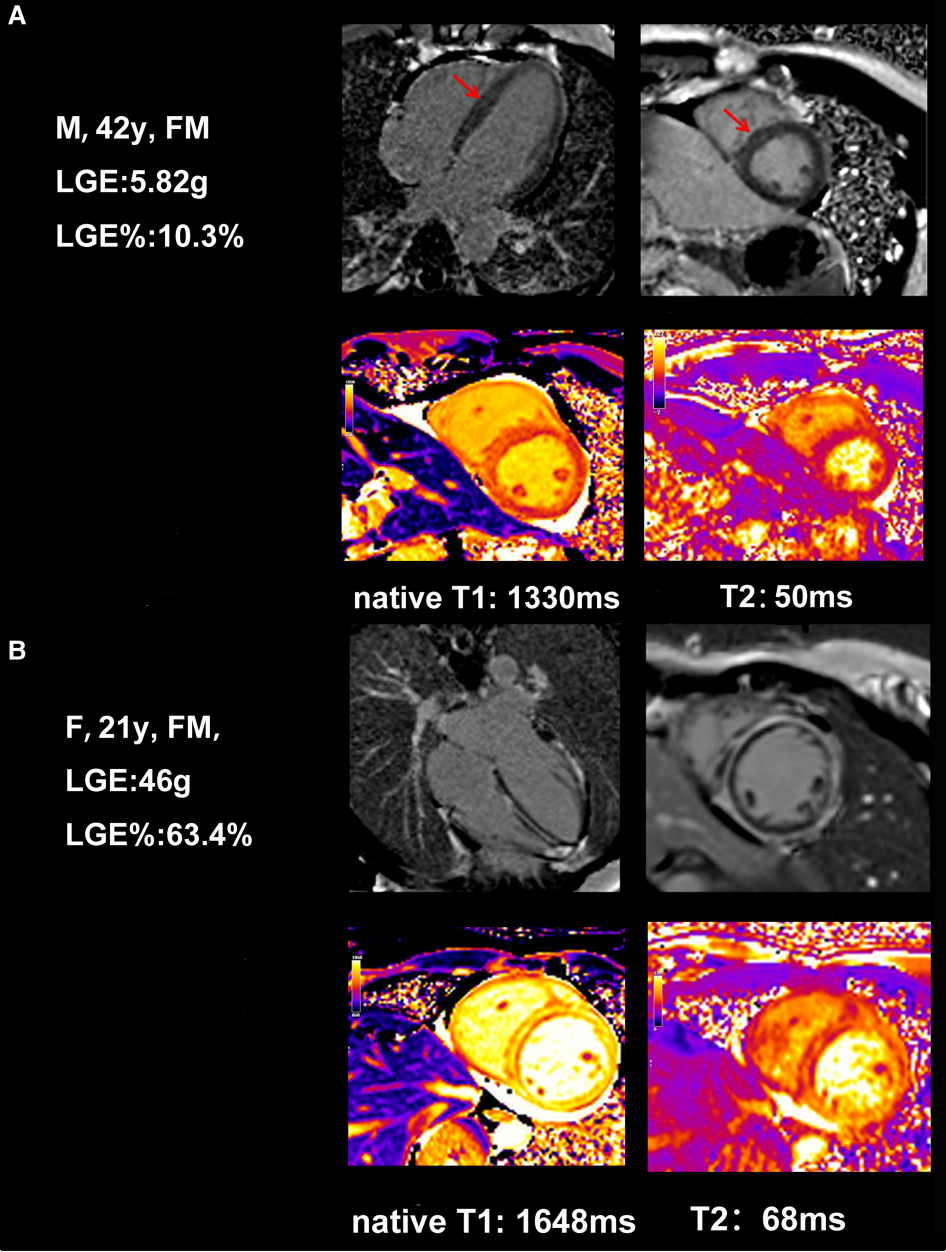
Example of typical cardiac MRI findings in a 42-year-old female FM patient with low LGE and a 21-year-old female FM patient with high LGE. (**A**) A 42-year-old woman with CMR short-axis and long-axis view with septal LGE (arrows) and global native T1, T2. (**B**) A 21-year-old woman with CMR short-axis and long-axis view with diffuse and a large amount of LGE in the intermediolateral segments, and global native T1, T2.

Because of their critical condition, CMR examination was less feasible and often delayed in patients with FM. The comparison of CMR between patients in these two groups at discharge is demonstrated in Table 2. Mean time from admission to CMR showed no difference between the two groups [12 (10.5–16.5) days vs. 14 (10–17) days, *P* = 0.715]. CMR sequences suggestive of LGE were found in all patients; however, LGE mass% varies among FM patients, which can categorize them into low and high LGE groups (9.46% ± 6.29% vs. 46.48% ± 17.44%, *P* < 0.001). Table 2 also shows that the values of myocardial native T1 and T2 in the high LGE group were significantly higher than those in the low LGE group (*P* = 0.002). The CMR images of FM patients with different LGE patterns are shown in Figure 3. There were no significant differences between the low LGE group and the high LGE group of other conventional CMR parameters.

**Table 2:** Comparison of CMR between FM patients with low LGE and high LGE.

	Low LGE	High LGE	*P*
Heart rate (/min)	73.07 ± 11.56	73.14 ± 13.15	0.987
Day in hospitalization	12 (10.5–16.5)	14 (10–17)	0.715
Mean time to CMR	7.71 ± 2.61	8.78 ± 3.59	0.269
Cardiac morphology and function			
LVEF (%)	58.29 ± 10.45	54.29 ± 12.51	0.331
LVEDV (ml)	122.86 ± 30.64	112.76 ± 36.39	0.399
LVEDVi (ml/m^2^)	72.43 ± 14.47	67.24 ± 20.67	0.421
LVESV (ml)	53.21 ± 24.56	49.90 ± 18.68	0.654
LVESVi (ml/m^2^)	31.00 ± 13.10	39.05 ± 9.91	0.619
LVMi (g/m^2^)	67.73 ± 18.45	62.88 ± 20.87	0.486
CO (I/min)	5.10 ± 1.22	5.14 ± 1.49	0.935
SV (ml)	69.57 ± 11.77	62.86 ± 21.43	0.294
Myocardial tissue characterization			
Native T1 (ms)	1,324.00 ± 56.52	1,456.77 ± 105.09	0.002
T2 (ms)	44.00 (42.75–45.00)	49.00 (46.50–58.00)	0.002
LGE mass (g)	6.48 ± 4.77	28.00 ± 17.23	<0.001
LGE mass%	9.46 ± 6.29	46.48 ± 17.44	<0.001

LVEF, left ventricular ejection fraction; LVEDVi, left ventricular end-diastolic volume index; LVESVi, left ventricular end-systolic volume index; LVMi, left ventricular mass index; CO, cardiac output; SV, stroke volume; LGE, late gadolinium enhancement.

### 3.3. Conventional echocardiographic and strain analysis

**Figure 4:**
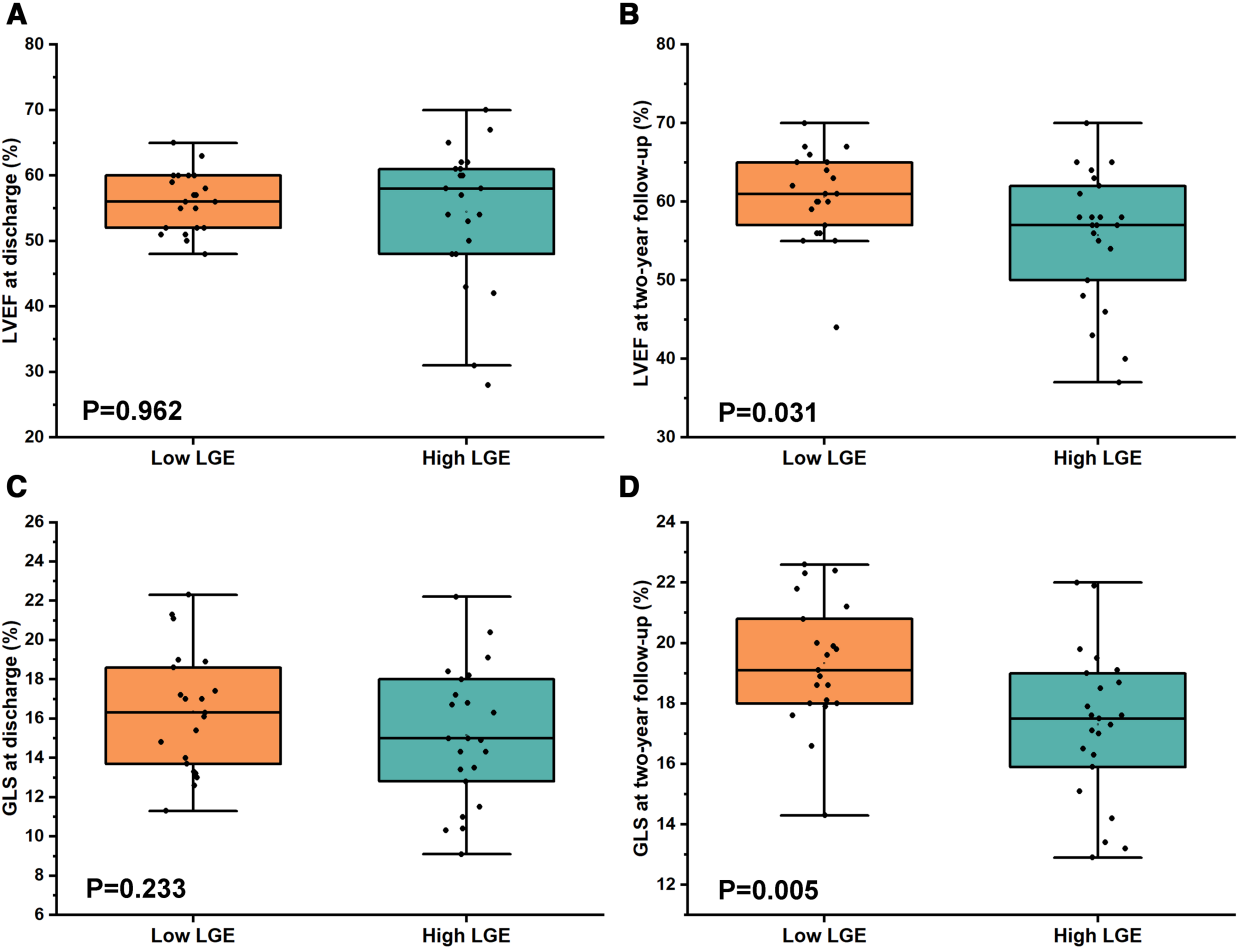
Comparison of LVEF and GLS between low LGE and high LGE at discharge and at 2-year follow-up in FM patients. (**A,C**) No significant difference for both LVEF [52.00 (56.00–60.00) vs. 58.00 (49.00–61.50), *P* = 0.962] and GLS (16.36 ± 3.08 vs. 15.17 ± 3.42, *P* = 0.233) between low LGE and high LGE at discharge. (**B,D**) However, there was a significant difference for both LVEF (60.62 ± 5.72 vs. 55.74 ± 8.37, *P* = 0.031) and GLS (19.34 ± 2.07 vs. 17.30 ± 2.47, *P* = 0.005) between low LGE and high LGE at 2-year follow-up.

Conventional echocardiographic and strain measurements between the low LGE group and the high LGE group at discharge are shown in Table 3. The data of conventional echocardiography between two patterns patients at admission are shown in Supplementary Table S2. There was no significant difference between the two groups at discharge for the LVEDD (4.80 ± 0.47 cm vs. 4.67 ± 0.59 cm, *P* = 0.426), LVEF [52.00% (56.00%–60.00%) vs. 58.00% (49.00%–61.50%), *P* = 0.962], and GLS (16.36% ± 3.08% vs. 15.17% ± 3.42%, *P* = 0.233, Figures 4A,C).

**Table 3:** Comparison of echocardiographic parameters between low LGE and high LGE at discharge.

Parameters	Low LGE	High LGE	*P*
IVS D (cm)	0.94 ± 0.17	0.96 ± 0.18	0.797
IVS S (cm)	1.24 ± 0.16	1.22 ± 0.22	0.837
LVEDD (cm)	4.80 ± 0.47	4.67 ± 0.59	0.426
LVEDS (cm)	3.34 ± 0.42	3.53 ± 0.64	0.278
End-diastolic volume (ml)	115.00 ± 27.20	122.47 ± 34.17	0.464
End-systolic volume (ml)	53.05 ± 16.28	57.18 ± 22.65	0.524
LA D (cm)	3.30 ± 0.55	3.25 ± 0.56	0.632
LV ejection fraction (%)	52.00 (56.00–60.00)	58.00 (49.00–61.50)	0.962
E-wave deceleration time (cm/sec)	77.10 ± 18.62	84.86 ± 27.84	0.411
E/A	1.23 (1.00–1.58)	1.56 (1.11–2.00)	0.17
E’	8.43 ± 2.31	8.80 ± 2.59	0.966
E/E’	9.19 (8.55–10.49)	10.00 (8.65–11.96)	0.204
GLS	16.36 ± 3.08	15.17 ± 3.42	0.233

IVS, interventricular septum; LVEDD, end-diastolic dimensions; LVESD, end-systolic dimensions; LA, left atrium; EF, ejection fraction; E, peak early diastolic mitral flow velocity; A, peak late diastolic mitral flow velocity; E’, spectral pulsed-wave Doppler–derived early diastolic velocity from the septal mitral annulus; GLS, global peak systolic longitudinal strain.

The results for echocardiographic and strain parameters between the two groups at 2-year follow-up are shown in Table 4. In comparison with high LGE groups, the LVEF (60.62% ± 5.72% vs. 55.74% ± 8.37%, *P* = 0.031) and the GLS (19.34% ± 2.07% vs. 17.30% ± 2.47%, *P* = 0.005, Figures 4B,D) at 2-year follow-up were significantly higher in the low LGE group.

**Table 4:** Comparison of echocardiographic parameters between low LGE and high LGE at 2-year follow-up.

Parameters	Low LGE	High LGE	*P*
IVS D (cm)	0.90 (0.75–1.0)	0.80 (0.70–1.0)	0.375
IVS S (cm)	1.20 ± 0.24	1.03 ± 0.23	0.031
LVEDD (cm)	4.50 (4.40–4.88)	4.60 (4.30–5.10)	0.538
LVEDS (cm)	3.30 (2.93–3.55)	3.20 (2.90–3.90)	0.767
End-diastolic volume (ml)	115.76 ± 32.27	115.85 ± 30.86	0.993
End-systolic volume (ml)	46.00 (34.25–37.00)	43.00 (37.00–67.00)	0.695
LA D (cm)	3.24 ± 0.41	3.16 ± 0.34	0.512
LV ejection fraction (%)	60.62 ± 5.72	55.74 ± 8.37	0.031
E-wave deceleration time (cm/sec)	69.95 ± 17.09	79.55 ± 18.77	0.095
E/A	1.17 (0.93–1.52)	1.58 (1.25–1.96)	0.022
E’	9.14 ± 2.59	8.80 ± 2.07	0.643
E/E’	8.23 (6.77–9.47)	8.78 (7.49–10.16)	0.13
GLS (%)	19.34 ± 2.07	17.30 ± 2.47	0.005

IVS, interventricular septum; LVEDD, end-diastolic dimensions; LVESD, end-systolic dimensions; LA, left atrium; EF, ejection fraction; E, peak early diastolic mitral flow velocity; A, peak late diastolic mitral flow velocity; E’, spectral pulsed-wave Doppler–derived early diastolic velocity from the septal mitral annulus; GLS, global peak systolic longitudinal strain.

**Figure 5:**
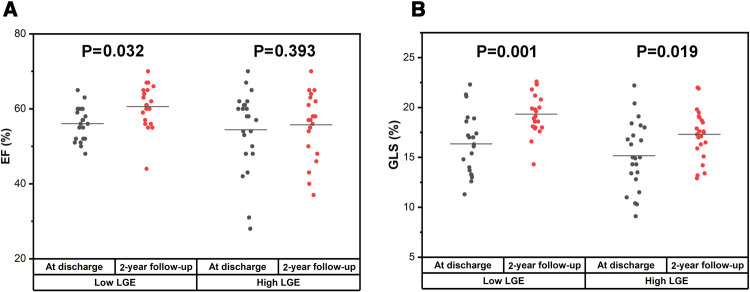
Comparison of LVEF and GLS between at discharge and at 2-year follow-up in the low LGE group and the high LGE group. (**A**) There was a significant improvement for LVEF in the low LGE group [52.00% (56.00%–60.00%) vs. 61.00% (56.50%–65.00%), *P* = 0.032] but not for the high LGE group [58.00% (48.00%–61.00%) vs. 57.00% (50.00%–62.00%), *P* = 0.393]. (**B**) The GLS at 2-year follow-up was significantly higher than that at discharge for both groups (16.36 ± 3.08 vs. 19.34 ± 2.07, *P* = 0.001 and 15.17 ± 3.42 vs. 17.30 ± 2.47, *P* = 0.019).

There was no significant improvement of LVEF in the high LGE group at 2-year follow-up compared with that at discharge [58.00% (48.00%–61.00%) vs. 57.00% (50.00%–62.00%), *P* = 0.393, Figure 5A]. However, the LVEF was significantly elevated at 2-year follow-up in the low LGE group [52.00% (56.00%–60.00%) vs. 61.00% (56.50%–65.00%), *P* = 0.032, Figure 5A]. In contrast to LVEF, the GLS at 2-year follow-up was significantly higher than those at discharge in both two groups (16.36 ± 3.08 vs. 19.34 ± 2.07 in the low LGE group, *P* = 0.001 and 15.17 ± 3.42 vs. 17.30 ± 2.47 in the high LGE group, *P* = 0.019, Figure 5B).

### 3.4. Correlation of echocardiographic and CMR parameters of patients with FM

**Figure 6:**
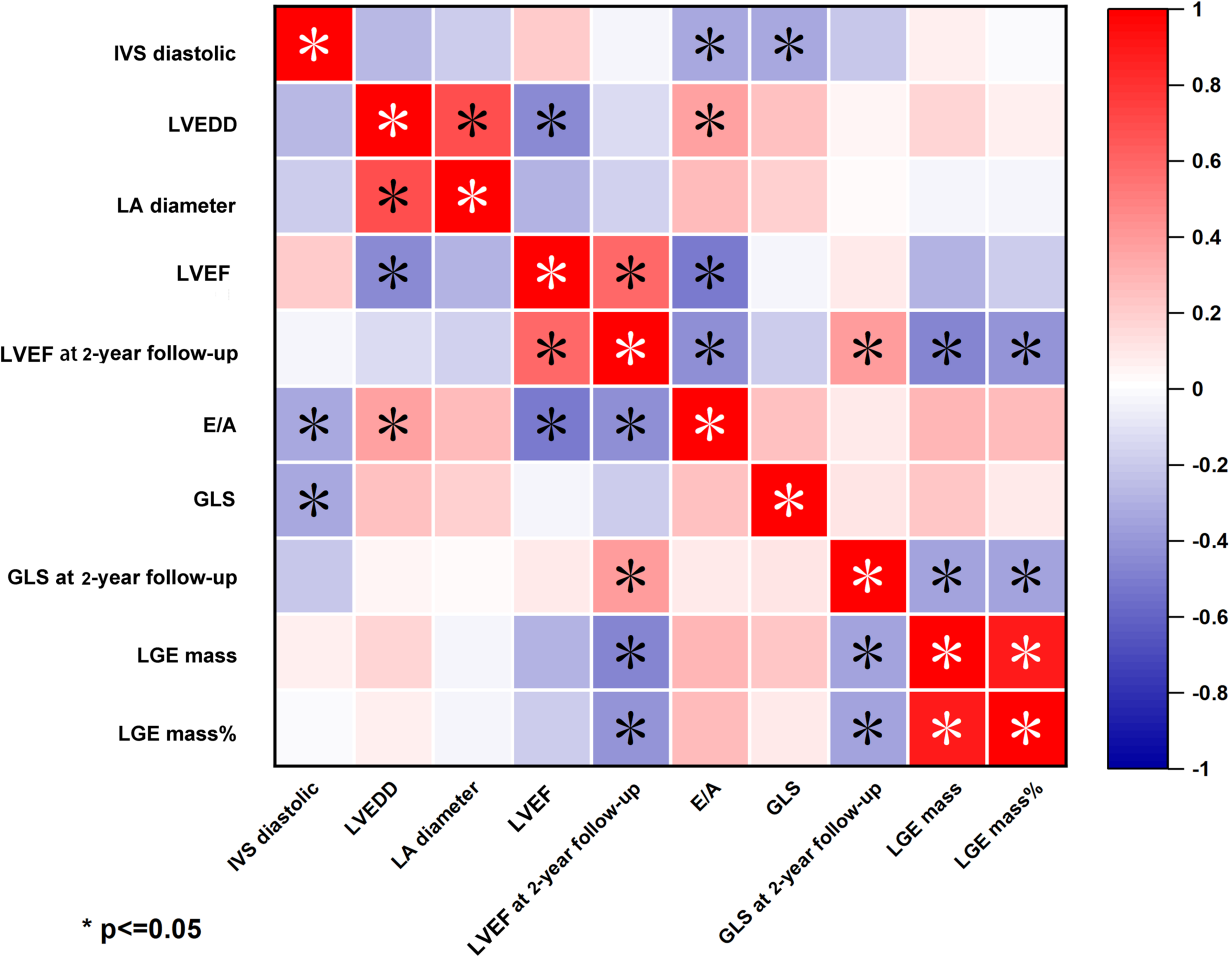
Heatmap of correlation between echocardiography parameters and LGE-CMR in fulminant myocarditis. LGE mass% was negative correlation with LVEF and GLS at 2-year follow-up.

Taking the whole population into consideration, we next analyzed the correlation between echocardiographic indices and CMR parameters. We found that LGE mass% negatively correlated with LVEF and GLS both at discharge and 2-year follow-up. (LVEF at 2-year follow-up: *r* = −0.437, *P* = 0.003; GLS at 2-year follow-up: *r* = −0.439, *P* = 0.003, Figure 6). However, there were no significant correlations between LGE mass% at discharge and hs-TNT at discharge, as was in LGE mass (g) (Supplementary Figure S1).

## 4. Discussion

In this study, we assessed the cardiac function of two groups of patients with FM at the 2-year follow-up. The main results of the present study are as follows: (1) two main patterns of LGE were found in patients with FM: low LGE and high LGE; (2) the high LGE group had worse cardiac function at 2-year follow-up than the low LGE group; (3) recovery of cardiac function was not significant in the high LGE group at the 2-year follow-up; and (4) the LGE mass% at discharge was negatively correlated with LVEF and GLS at the 2-year follow-up.

### 4.1. Two patterns of LGE presence in FM patients

Previous studies of patients with myocarditis revealed that LGE was higher in FM patients than in NFAM patients (25); however, there was a scarcity of data about the LGE patterns in patients with FM. In this study, patients with FM were separated into two groups: low LGE and high LGE, based on whether the LGE mass% at discharge was greater than 20.

It has been histologically proven that LGE represents areas of myocardial fibrosis and irreversible myocardial necrosis (26, 27), which means more inflammation and fibrosis, leading to a worse prognosis. Therefore, we hypothesized that the low LGE group had less myocardial necrosis and less inflammation, whereas the high LGE group had predominant myocardial necrosis and more severe edema. We also tested the level of hs-TNT and NT-proBNP, biomarkers of myocardial injury, and heart failure to reflect the degree of myocardial necrosis. Our result suggested that compared with the low LGE group, the peak hs-TNT and NT-proBNP at discharge was higher in the high LGE group. Moreover, compared with the low LGE group, the high LGE group has increased native T1 values (representing diffuse fibrosis or inflammation) and increased T2 values (suggesting diffuse myocardial inflammation). To a certain extent, this result confirms our assumptions: there are two different patterns of LGE in patients with FM. Myocardial necrosis and diffuse inflammation was more present in the high LGE group, while the low LGE group had less myocardial edema and necrosis.

We speculate that the different patterns of LGE may be associated with different strains of viral infections (28) and pathological typing. For example, Herpesvirus 6 infects not only T cells but also the cardiac conduction system and nervous system, resulting in decreased myocardial contractility and malignant arrhythmias (29). In 2019, Ammirati et al. revealed that (30) patients with histologically proven eosinophilic myocarditis, lymphocytic myocarditis, and giant-cell myocarditis all exhibited poor prognosis. Nevertheless, patients with giant-cell myocarditis had a strikingly higher rate of early death or heart transplantation compared with eosinophilic myocarditis and lymphocytic myocarditis.

### 4.2. The comparison of cardiac function between high LGE and low LGE

LGE-MRI allows noninvasive assessment of fibrosis and scar, playing an increasingly important role in the depiction of myocardial fibrosis (31–33). In LGE-MRI, imaging is performed 15–20 min after injection of a gadolinium-based contrast agent, which allows the contrast distributing to the extravascular-extracellular spaces and accumulating in regions of fibrosis.

Recent studies have shown that LGE assessed by CMR is an outcome predictor of future adverse cardiac events in patients with myocarditis (14, 16, 34). Grani et al. demonstrated that LGE extent (every 10% increase) was a predictor of higher risk of major adverse cardiac events (14). Additionally, LGE of 20% of LV mass confers almost twofold increase in sudden cardiac death risk in patients with hypertrophic cardiomyopathy (35). So far, several studies have revealed the high specificity of LGE for the detection of myocardial injury in myocarditis (36, 37). However, the data on LGE in patients with fulminant myocarditis remains challenging because of the limited number of patients. Only Li et al. showed that patients with FM had significant differences in LGE patterns compared to NFAM patients (25). Our study found that the high LGE group had worse cardiac function than those with the low LGE group at 2-year follow-up, although there was no significant difference at discharge. Our findings from the present study are in line with the 2014 study by Schumm et al. and support that myocarditis with higher LGE is worse in prognosis (38).

### 4.3. The comparison of cardiac function between at discharge and at 2-year follow-up

In the present study, our analysis suggests the significant recovery of LVEF and GLS at 2-year follow-up compared with those at discharge in the low LGE group, whereas no difference of LVEF in the high LGE group between at 2-year follow-up and at discharge was observed. However, results from the GLS are different, compared to the value at discharge; the GLS at 2-year follow-up was significantly higher in both groups. Our data indicate that the GLS of FM patients improved regardless of the LGE but only the low LGE group showed an improvement in LVEF, while the changes of LVEF in the high LGE group is not appreciable. That is probably because strain imaging represents a more valuable tool to reveal subtle changes of cardiac function (39) during follow-up than LVEF. It also reflected the better recovery of FM patients in the low LGE group (with less scarring). Aquaro et al. (34) showed that the presence of a myocardial scar is generally associated with increased risk for adverse cardiovascular events, even in patients with ejection fraction (EF) >50%. LGE is widely accepted for detecting myocardial fibrosis and all forms of irreversible myocardial injury, such as myocardial infarction (40), and is correlated to LV remodeling (28). These were in fact consistent with our result that the FM patients with higher LGE extent had poor recovery of cardiac function. Moreover, fibrosis is associated with contractile impairment and provides a structural basis for ventricular reentrant arrhythmia (41, 42). In our study, more patients in the high LGE group [13 of 23 (56.5%)] showed ST-segment elevation during hospitalization than the low LGE group (4 of 21, 19%; *P* = 0.001) in.

### 4.4. The correlation between LGE and echocardiography parameters

The meta-analysis by Georgiopoulos et al. (43) found that the risk of experiencing the combined endpoint was doubled in patients with more extensive LGE (i.e., >2 LV segments with LGE or LGE > 10% of LV mass or LGE > 17 g) as compared with those with small or no LGE burden. However, in our study, no death occurred during hospitalization in both two groups. The possible reason may be attributed to the LSBCTR applied in our center, which successfully lowered in-hospital mortalities of FM to <4%. In the present study, LGE mass% at discharge showed a negative correlation with LV function at 2-year follow-up detected by LVEF and GLS. Other previous studies also demonstrated that some parameters could predict outcomes or LV functional recovery after discharge in myocarditis. Rodriguez-Gonzalez et al. revealed that the presence of LVEF <30% at admission was the major predictor of poor outcomes in children with myocarditis (44). However, Ammirati et al. did not suggest the prognostic value of LVEF (30).

### 4.5. Limitation

First, this was a single-center study with relatively small sample size. Thus, the selection bias was present and the conclusion drawn from the study must be considered in the context of the small study population. Next, we did not perform the EMB in all patients. However, the diagnosis of myocarditis in the study was made by the clinical data as elevated troponin level in combination with typical CMR findings of myocarditis, which were common in the clinical practice of the real world and also in many reports of FM (45). However, due to the absence of EMB data, it was not known whether the results obtained from this study population were different or not for various histological type of FM. Finally, this was an observational study including only FM patients. No data from NFAM were included to make a comparison between FM and NFAM, which may also affect the reliability of the study.

Overall, future study with larger sample size and longer follow-up period is needed.

## 5. Conclusions

Our data demonstrate the two distinct patterns of LGE presentation in patients with FM. The cardiac function of the high LGE group was significantly worse than those in the low LGE group at 2-year follow-up. Moreover, the recovery of cardiac function in the high LGE group was not significant during the 2-year follow-up. LGE mass% at discharge was negatively correlated with LVEF and GLS at 2-year follow-up.

## Data Availability

The original contributions presented in the study are included in the article/Supplementary Material, further inquiries can be directed to the corresponding authors.

## References

[1] Fuse K Kodama M Okura Y Ito M Hirono S Kato K Predictors of disease course in patients with acute myocarditis. Circulation. (2000) 102:2829–35. 10.1161/01.CIR.102.23.2829 11104740

[2] Basso C. Myocarditis. N Engl J Med. (2022) 387:1488–500. 10.1056/NEJMra2114478 36260793

[3] Zhou N Zhao Y Jiang J Shen L Li J Wan J Impact of mechanical circulatory support and immunomodulation therapy on outcome of patients with fulminant myocarditis: Chinese registry of fulminant myocarditis. Signal Transduct Target Ther. (2021) 6:350. 10.1038/s41392-021-00700-6 34611131 PMC8492670

[4] Diddle JW Almodovar MC Rajagopal SK Rycus PT Thiagarajan RR. Extracorporeal membrane oxygenation for the support of adults with acute myocarditis. Crit Care Med. (2015) 43:1016–25. 10.1097/CCM.0000000000000920 25738858

[5] Rodriguez A Alvarez-Rocha L Sirvent JM Zaragoza R Nieto M Arenzana A Recommendations of the Infectious Diseases Work Group (GTEI) of the Spanish Society of Intensive and Critical Care Medicine and Coronary Units (SEMICYUC) and the Infections in Critically Ill Patients Study Group (GEIPC) of the Spanish Society of Infectious Diseases and Clinical Microbiology (SEIMC) for the diagnosis and treatment of influenza A/H1N1 in seriously ill adults admitted to the intensive care unit. Med Intensiva. (2012) 36:103–37. 10.1016/j.medin.2011.11.020 22245450

[6] Schubert S Opgen-Rhein B Boehne M Weigelt A Wagner R Muller G Severe heart failure and the need for mechanical circulatory support and heart transplantation in pediatric patients with myocarditis: results from the prospective multicenter registry “MYKKE”. Pediatr Transplant. (2019) 23:e13548. 10.1111/petr.13548 31297930

[7] Li S Xu S Li C Ran X Cui G He M A life support-based comprehensive treatment regimen dramatically lowers the in-hospital mortality of patients with fulminant myocarditis: a multiple center study. Sci China Life Sci. (2019) 62:369–80. 10.1007/s11427-018-9501-9 30850929

[8] Kanaoka K Onoue K Terasaki S Nakano T Nakai M Sumita Y Features and outcomes of histologically proven myocarditis with fulminant presentation. Circulation. (2022) 146:1425–33. 10.1161/CIRCULATIONAHA.121.058869 36164974

[9] Chow LH Radio SJ Sears TD McManus BM. Insensitivity of right ventricular endomyocardial biopsy in the diagnosis of myocarditis. J Am Coll Cardiol. (1989) 14:915–20. 10.1016/0735-1097(89)90465-8 2794278

[10] Hauck AJ Kearney DL Edwards WD. Evaluation of postmortem endomyocardial biopsy specimens from 38 patients with lymphocytic myocarditis: implications for role of sampling error. Mayo Clin Proc. (1989) 64:1235–45. 10.1016/S0025-6196(12)61286-5 2593714

[11] Baughman KL. Diagnosis of myocarditis: death of Dallas criteria. Circulation. (2006) 113:593–5. 10.1161/CIRCULATIONAHA.105.589663 16449736

[12] Bozkurt B Colvin M Cook J Cooper LT Deswal A Fonarow GC Current diagnostic and treatment strategies for specific dilated cardiomyopathies: a scientific statement from the American Heart Association. Circulation. (2016) 134:e579–646. 27832612 10.1161/CIR.0000000000000455

[13] Luetkens JA Doerner J Thomas DK Dabir D Gieseke J Sprinkart AM Acute myocarditis: multiparametric cardiac MR imaging. Radiology. (2014) 273:383–92. 10.1148/radiol.14132540 24910904

[14] Grani C Eichhorn C Biere L Murthy VL Agarwal V Kaneko K Prognostic value of cardiac magnetic resonance tissue characterization in risk stratifying patients with suspected myocarditis. J Am Coll Cardiol. (2017) 70:1964–76. 10.1016/j.jacc.2017.08.050 29025553 PMC6506846

[15] Maron MS. Clinical utility of cardiovascular magnetic resonance in hypertrophic cardiomyopathy. J Cardiovasc Magn Reson. (2012) 14:13. 10.1186/1532-429X-14-13 22296938 PMC3293092

[16] Grun S Schumm J Greulich S Wagner A Schneider S Bruder O Long-term follow-up of biopsy-proven viral myocarditis: predictors of mortality and incomplete recovery. J Am Coll Cardiol. (2012) 59:1604–15. 10.1016/j.jacc.2012.01.007 22365425

[17] Barone-Rochette G Augier C Rodiere M Quesada JL Foote A Bouvaist H Potentially simple score of late gadolinium enhancement cardiac MR in acute myocarditis outcome. J Magn Reson Imaging. (2014) 40:1347–54. 10.1002/jmri.24504 24293405

[18] Wang H Zhao B Jia H Gao F Zhao J Wang C. A retrospective study: cardiac MRI of fulminant myocarditis in children—can we evaluate the short-term outcomes? Peer J. (2016) 4:e2750. 10.7717/peerj.2750 27994968 PMC5162402

[19] Mavrogeni S Bratis K Terrovitis J Tsagalou E Nanas J. Fulminant myocarditis. Can cardiac magnetic resonance predict evolution to heart failure? Int J Cardiol. (2012) 159:e37–8. 10.1016/j.ijcard.2011.11.053 22209570

[20] Ryu DR Heo JW Lee SH Lee W Choi JW Kim HY Fulminant myocarditis: the role of cardiac magnetic resonance imaging. Int J Cardiol. (2013) 168:e58–9. 10.1016/j.ijcard.2013.07.002 23871628

[21] Ammirati E Cipriani M Moro C Raineri C Pini D Sormani P Clinical presentation and outcome in a contemporary cohort of patients with acute myocarditis: multicenter Lombardy registry. Circulation. (2018) 138:1088–99. 10.1161/CIRCULATIONAHA.118.035319 29764898

[22] Lang RM Badano LP Mor-Avi V Afilalo J Armstrong A Ernande L Recommendations for cardiac chamber quantification by echocardiography in adults: an update from the American Society of Echocardiography and the European Association of Cardiovascular Imaging. J Am Soc Echocardiogr. (2015) 28:1–39.e14. 10.1016/j.echo.2014.10.003 25559473

[23] Zuo H Li H Li R Ma F Jiang J Li C Myocardial strain features by 2D-STE during the course of fulminant myocarditis: correlation with characteristics by CMR and clinical implications. Medicine. (2021) 100:e25050. 10.1097/MD.0000000000025050 33847613 PMC8052038

[24] Maron MS. Contrast-enhanced CMR in HCM: what lies behind the bright light of LGE and why it now matters. JACC Cardiovasc Imaging. (2013) 6:597–9. 10.1016/j.jcmg.2012.10.028 23582359

[25] Li H Zhu H Yang Z Tang D Huang L Xia L. Tissue characterization by mapping and strain cardiac MRI to evaluate myocardial inflammation in fulminant myocarditis. J Magn Reson Imaging. (2020) 52:930–8. 10.1002/jmri.27094 32080960

[26] Moon JC Sachdev B Elkington AG McKenna WJ Mehta A Pennell DJ Gadolinium enhanced cardiovascular magnetic resonance in Anderson-Fabry disease. Evidence for a disease specific abnormality of the myocardial interstitium. Eur Heart J. (2003) 24:2151–5. 10.1016/j.ehj.2003.09.017 14643276

[27] Gange CA Link MS Maron MS. Utility of cardiovascular magnetic resonance in the diagnosis of Anderson-Fabry disease. Circulation. (2009) 120:e96–7. 10.1161/CIRCULATIONAHA.109.849828 19786638

[28] Mahrholdt H Wagner A Deluigi CC Kispert E Hager S Meinhardt G Presentation, patterns of myocardial damage, and clinical course of viral myocarditis. Circulation. (2006) 114:1581–90. 10.1161/CIRCULATIONAHA.105.606509 17015795

[29] Prober C. Sixth disease and the ubiquity of human herpesviruses. N Engl J Med. (2005) 352:753–5. 10.1056/NEJMp048302 15728806

[30] Ammirati E Veronese G Brambatti M Merlo M Cipriani M Potena L Fulminant versus acute nonfulminant myocarditis in patients with left ventricular systolic dysfunction. J Am Coll Cardiol. (2019) 74:299–311. 10.1016/j.jacc.2019.04.063 31319912

[31] Kim RJ Wu E Rafael A Chen EL Parker MA Simonetti O The use of contrast-enhanced magnetic resonance imaging to identify reversible myocardial dysfunction. N Engl J Med. (2000) 343:1445–53. 10.1056/NEJM200011163432003 11078769

[32] Simonetti OP Kim RJ Fieno DS Hillenbrand HB Wu E Bundy JM An improved MR imaging technique for the visualization of myocardial infarction. Radiology. (2001) 218:215–23. 10.1148/radiology.218.1.r01ja50215 11152805

[33] Caspar T Fichot M Ohana M El Ghannudi S Morel O Ohlmann P. Late detection of left ventricular dysfunction using two-dimensional and three-dimensional speckle-tracking echocardiography in patients with history of nonsevere acute myocarditis. J Am Soc Echocardiogr. (2017) 30:756–62. 10.1016/j.echo.2017.04.002 28599827

[34] Aquaro GD Perfetti M Camastra G Monti L Dellegrottaglie S Moro C Cardiac MR with late gadolinium enhancement in acute myocarditis with preserved systolic function: ITAMY study. J Am Coll Cardiol. (2017) 70:1977–87. 10.1016/j.jacc.2017.08.044 29025554

[35] Weng Z Yao J Chan RH He J Yang X Zhou Y Prognostic value of LGE-CMR in HCM: a meta-analysis. JACC Cardiovasc Imaging. (2016) 9:1392–402. 10.1016/j.jcmg.2016.02.031 27450876

[36] Ferreira VM Schulz-Menger J Holmvang G Kramer CM Carbone I Sechtem U Cardiovascular magnetic resonance in nonischemic myocardial inflammation: expert recommendations. J Am Coll Cardiol. (2018) 72:3158–76. 10.1016/j.jacc.2018.09.072 30545455

[37] Mahrholdt H Goedecke C Wagner A Meinhardt G Athanasiadis A Vogelsberg H Cardiovascular magnetic resonance assessment of human myocarditis: a comparison to histology and molecular pathology. Circulation. (2004) 109:1250–8. 10.1161/01.CIR.0000118493.13323.81 14993139

[38] Schumm J Greulich S Wagner A Grun S Ong P Bentz K Cardiovascular magnetic resonance risk stratification in patients with clinically suspected myocarditis. J Cardiovasc Magn Reson. (2014) 16:14. 10.1186/1532-429X-16-14 24461053 PMC3913958

[39] Meindl C Paulus M Poschenrieder F Zeman F Maier LS Debl K. Patients with acute myocarditis and preserved systolic left ventricular function: comparison of global and regional longitudinal strain imaging by echocardiography with quantification of late gadolinium enhancement by CMR. Clin Res Cardiol. (2021) 110:1792–800. 10.1007/s00392-021-01885-0 34086089 PMC8563632

[40] Zagrosek A Abdel-Aty H Boye P Wassmuth R Messroghli D Utz W Cardiac magnetic resonance monitors reversible and irreversible myocardial injury in myocarditis. JACC Cardiovasc Imaging. (2009) 2:131–8. 10.1016/j.jcmg.2008.09.014 19356545

[41] Iles L Pfluger H Lefkovits L Butler MJ Kistler PM Kaye DM Myocardial fibrosis predicts appropriate device therapy in patients with implantable cardioverter-defibrillators for primary prevention of sudden cardiac death. J Am Coll Cardiol. (2011) 57:821–8. 10.1016/j.jacc.2010.06.062 21310318

[42] Rubinshtein R Glockner JF Ommen SR Araoz PA Ackerman MJ Sorajja P Characteristics and clinical significance of late gadolinium enhancement by contrast-enhanced magnetic resonance imaging in patients with hypertrophic cardiomyopathy. Circ Heart Fail. (2010) 3:51–8. 10.1161/CIRCHEARTFAILURE.109.854026 19850699

[43] Georgiopoulos G Figliozzi S Sanguineti F Aquaro GD di Bella G Stamatelopoulos K Prognostic impact of late gadolinium enhancement by cardiovascular magnetic resonance in myocarditis: a systematic review and meta-analysis. Circ Cardiovasc Imaging. (2021) 14:e011492. 10.1161/CIRCIMAGING.120.011492 33441003

[44] Rodriguez-Gonzalez M Sanchez-Codez MI Lubian-Gutierrez M Castellano-Martinez A. Clinical presentation and early predictors for poor outcomes in pediatric myocarditis: a retrospective study. World J Clin Cases. (2019) 7:548–61. 10.12998/wjcc.v7.i5.548 30863755 PMC6406197

[45] Pasupathy S Air T Dreyer RP Tavella R Beltrame JF. Systematic review of patients presenting with suspected myocardial infarction and nonobstructive coronary arteries. Circulation. (2015) 131:861–70. 10.1161/CIRCULATIONAHA.114.011201 25587100

