# Supplementary material for: The pattern of late gadolinium enhancement by cardiac MRI in fulminant myocarditis and its prognostic implication: a two-year follow-up study
Source: Front Cardiovasc Med. 2023 Jun 27;10:1144469. doi: 10.3389/fcvm.2023.1144469 (PMC10335322; doi:10.3389/fcvm.2023.1144469)
Supplement: Supplementary file 1 [file Datasheet1.pdf]

Supplementary data

Supplemental figure1: Dot plots of hs-TNT at discharge in relation to LGE mass （%） and LGE mass (g). There was no significant correlation for hs-TNT at discharge with LGE mass (%) nor LGE mass (g).

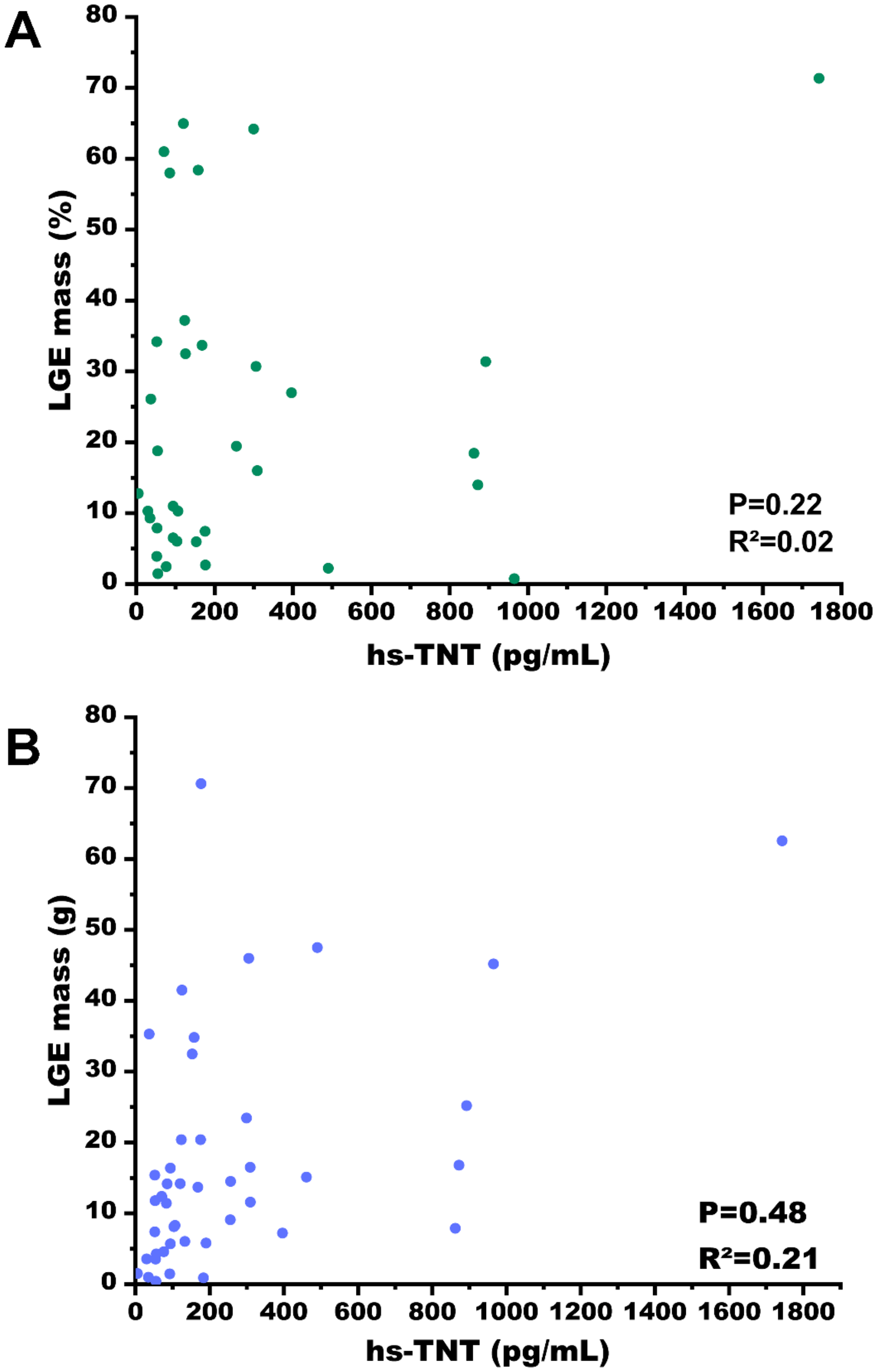

Table 1 Clinical Presentations of the low LGE and high LGE group in FM patients at admission

Clinical data	Low LGE	High LGE	P
Male -n(%)	11（52.38）	10（43.48）	0.555
Female -n(%)	10（47.62)	13（56.52）	0.555
Age(year)	33.71±12.48	31.83±11.46	0.604
Height(cm)	166.29±9.25	167.17±6.48	0.717
Weight(kg)	68.19±16.33	61.67±10.71	0.121
Systolic blood pressure(mmHg)	93.83±9.95	90.61±12.30	0.456
Diastolic blood pressure(mmHg)	58.92±7.97	59.06±9.35	0.967
Heart rate(bpm)	105.17±18.67	104.00±16.25	0.857
**Biochemistry Examination**			
CRP(mg/L)	13.45(4.68,70.58)	37.8(14.13,94.85)	0.262
Troponin-T(pg/mL)	29775.50(19491.75,48829.88)	49515.00(22493.00,50000.00)	0.267
NT-proBNP(pg/ml)	18750.89±14640.89	20702.33±16448.27	0.736
ALT(U/L)	83.50(66.25,189.00)	126.50(65.25,250.25)	0.491
AST(U/L)	132.50(118.25,228.50)	163.50(128.50,231.50)	0.325
Creatinine (μmol/L)	73.58±19.68	77.00±15.42	0.599
**Complication**			
hypertension	1 (4.76)	1 (4.35)	0.948
diabetes	1 (4.76)	2 (8.70)	0.601

ALT,alanine transaminase; AST,aspartate aminotransferase; NT-proBNP=N-terminal pro-B-type natriuretic peptide

Table2 Comparison of echocardiographic parameters between low LGE and high LGE at admission

Parameters	Low LGE	High LGE	P
IVS systolic (cm)	1.26±0.15	1.32±0.18	0.361
LVEDD (cm)	4.96±0.41	5.06±0.50	0.58
LA diameter (cm)	3.43±0.65	3.62±0.59	0.396
EF (%)	23.25±5.43	22.61±5.93	0.767

IVS, interventricular septum； LVEDD, LVend-diastolic dimensions;LA, left atrium; EF, ejection fraction
